# Histological Examination of Lemongrass Resorbable Dressing on Gingival Healing after Gingivectomy in Rats

**DOI:** 10.1055/s-0042-1748197

**Published:** 2022-06-27

**Authors:** Amaliya Amaliya, Indri Budhirahardjo, Ina Hendiani

**Affiliations:** 1 Departement of Periodontology, Dental Faculty, Universitas Padjadjaran, West Java, Indonesia; 2 Periodontal Clinic, Indonesian Naval Dental Institute Raden Eddy Martadinata, Central Jakarta

**Keywords:** lemongrass, dressing, wound healing, gingivectomy

## Abstract

**Objectives** The present work aimed to evaluate the effect of lemongrass extract incorporated in a resorbable periodontal dressing on gingival wound healing microscopically, following gingivectomy in rats.

**Materials and Methods** Thirty healthy adult male Sprague–Dawley rats were used in this study. Gingivectomy was performed on anterior area of lower jaw in the labial surface of central incisive and, subsequently, wound areas were covered with povidone iodine gel (group P, positive control, *n* = 10), lemongrass resorbable dressing (group L, *n* = 10), and a cellulose-based dressing containing myrrh (group M, positive control, *n* = 10). Histological changes were monitored in days 4, 7, and 14 postsurgery to evaluate fibroblast and collagen deposition as repair stage of healing process.

**Statistycal Analysis** One-way analysis of variance (ANOVA) followed by Tukey's post hoc for multiple comparisons were employed to measure differences between pairs of means, p-value of 0.05 was considered statistically significant.

**Results** We observed significant difference repair parameters of the healing process between surgical sites treated with lemongrass periodontal dressing and control groups. Wounds treated with lemongrass dressing had greater fibroblast compared with control groups in 4 and 7 days after surgery ( *p* ≤ 0.05).

**Conclusion** The results suggest positive potential therapeutic effects for this new formulation of periodontal dressing on acceleration of surgical wound healing that lead to improvement of periodontal treatment consequences following gingivectomy.

## Introduction

Oral cavity is a habitat of millions of pathogenic and opportunistic bacteria that are favorable for infections in the presence of any cut or wound in the epithelium. Surgical therapy performed in the oral cavity will result in oral wound, and without controlling the microbial activity, the wound healing delays and the treatment fails.

Periodontal pack and periodontal dressing are the terms used for periodontal bandages. To cover and protect wound surfaces, it is used after oral surgery, frequently applied around the necks of the teeth, and adjacent tissue. In earlier days, dressing acts as a protective barrier and has no curative properties. It is suggested to be used, as it helps in reducing discomfort and pain postoperatively by shielding the site of surgery even without any therapeutic effects. 1 On the other hand, several studies showed that the use of periodontal dressing has little beneficial effects, increases bacterial plaque accumulation around surgical sites, irritates healthy tissue, increases chance of infection and inflammation, and causes difficulty in eating. 2 3 Therefore, some of clinicians would prefer the wound site left without any dressings.

One of the surgical therapies frequently performed in dentistry is gingivectomy, defined as surgical removal of the gingiva to the bottom of the pocket. 4 It is indicated in cases of gingival enlargement where curettage is not able to reduce the excessive tissue. Nevertheless, gingivectomy using surgical blade or knife may create an extensive gingival wound and after the surgery is done, no sutures are placed, therefore the surgical sites should be covered to prevent postoperative infection and to accelerate healing. In this case, the role of periodontal dressings on gingival healing after gingivectomy is still of considerable interest to oral surgeons.

Many attempts have been made to enhance the properties of dressings, that is, incorporating chlorhexidine or antibiotics as antibacterial properties, to improve healing process and prevent infections. 5 6 Even though the addition of these agents is beneficial, possible problem may occur when antibiotics are involved, namely, emergence of resistant bacteria, opportunistic infections, the possibility of sensitization and allergy, and the potential development of candidiasis. 7 Therefore, alternatives of active compound should be discovered as a substitute and to avoid the adverse effect of antibiotics incorporated in dressings. Furthermore, it would be more favorable if the dressing will not serve as bacterial plaque retentive factor and do not interfere with mastication activity.

Among various types of periodontal pack, resorbable dressing offers advantages, since it does not require removal and can be safely degraded itself. It is a soft and soluble dressing that adheres to the wound surface, dissolves in approximately 30 hours and is consisted mostly of cellulose as a base which is a hydrophilic, elastic material with several active agents incorporated. 8 In this present study, we evaluate the effect of a resorbable pack with lemongrass extract as active ingredient. Lemongrass ( *Cymbopogon citratus* ) is a perennial herb belongs to *Poacea* , generically named as grasses. 9 Originating from the Southwest Asia, now it grows spontaneously around the world, especially in the tropical and savannah regions. 10 Lemongrass has been widely used as a traditional remedy by preparing the “tea” or infusion from fresh or dry leaves in almost all the continents and it comprises a wide range of indications, from mild conditions such as flu, fever, cuts, coughing, and headaches to more severe illness such as rheumatic, bladder disorder, diabetes, and malaria. 11 12 13 14 It is considered as a potent antibacterial, antitussive, antiseptic, analgesic, and anti-inflammatory agent. 15 16 17 18 The lemon-scented leaves of lemongrass is also used for insect repellent. 19 Studies discovering the use of lemongrass in dentistry showed that lemongrass oil prepared as mouthwash is effective in management of candidiasis, periodontitis, and as antidental plaque agent, and in the form of gel, it had been shown to reduce pocket in periodontitis patients. 20 21 22 23 24 However, little is known about the efficacy of lemongrass as active agent in periodontal dressing. We hypothesized that lemongrass wound dressing may enhance the healing process in terms of growth of fibroblasts and collagen deposition.

The present study aimed to evaluate the effect of lemongrass wound dressing on the healing process microscopically after gingivectomy in rats.

## Materials and Methods

This study evaluated and compared the effects of lemongrass periodontal dressing, povidone iodine gel, and a cellulose-based periodontal dressing with myrrh as active ingredient already available in the market (Reso-pac, Hager & Werken GmbH & Co. KG, Germany) on wound healing after gingivectomy in rats assessed by fibroblast proliferation and collagen deposition.

### Experimental Animals

In this study, 30 male Sprague–Dawley rats, weighing 250 to 350 g were recruited. One week before the experimental procedures, the animals underwent adaptation at air-conditioned animal laboratory room (22 ± 3°C) with 12-hour light and dark cycle. The rats were fed with commercial normal rodent pellet and filtered water ad libitum. All of the animals received humane care according to the criteria outlined in the Guide for the Care and the Use of Laboratory Animals prepared by the National Academy of Science and published by the National Institute of Health. 25 All of the experimental procedures were performed in Tropical Biopharmaca Research Centre, Bogor Agriculture Institute, West Java, Indonesia.

The experimental design and protocols were reviewed and approved by the Ethics Committee of the Experimental Animal Care Society, Biopharmaca Research Centre, Bogor Agriculture Institute, West Java, Indonesia, under file number 004–2017 KEH TROP BRC.

### Preparation of Lemongrass Ethanol Extract

Lemongrass was cultivated in Subang, West Java Province, Indonesia at 6° 34' 17.7204'' S 107° 45' 31.4496'' E, 700 m above sea level and collected in September 2016. Plant authentication was performed by a plant taxonomist with collection number BMK0204092016 deposited in Herbarium Bogoriense, Department of Biology, Bogor, West Java, Indonesia.

Fresh lemongrass of 5 kg was washed, minced, and dried in an oven for 3 days at 50°C. Dried leaves were then ground to powder. Maceration of lemongrass was performed by soaking powder in 70% ethanol, with a ratio of 1:10 for 3 × 24 hours at room temperature (±27°C) and stirred with a shaker at 150 rpm, then filtered and concentrated by rotary evaporator at a temperature of 50 to 60°C. Ethanol extract obtained was dark green in color and tasted bitter.

### Preparation of Lemongrass Resorbable Dressing

Dressing was prepared using thick extract of lemongrass, propylene glycol, methyl paraben, prophyl paraben, aquadest, and hydroxypropilmethyl cellulose (HPMC) as gelling agent. HPMC was soaked in hot aquadest (80°C) for a period of 2 hours. The dispersion was then allowed to hydrate and swelled for 15 minutes. Subsequently, methyl paraben and prophyl paraben were dissolved in ethanol and were added to swollen HPMC. Thick extract of lemongrass was then poured into the mixture, and then prophylene glycol was added and stirred until a homogenous gel was achieved. Aquadest was added until the mixture form a gel base.

### Gingivectomy and Experimental Animal Groups

Sample size calculation was performed by using the following formula:

( *r* – 1) ( *t* – 1) ≥ 15

Where, *t* = number of groups

*r* = number of samples

(3–1)( *n* – 1) ≥ 15

2 *n* – 2 ≥ 15

*n* ≥ 8.5

The minimum sample size per group was 8.5 rounded to 9. To anticipate drop out, 9 was rounded to 10.

The animals were randomly divided into three experimental groups ( *n* = 10 per group). These groups received treatments as follows:

- Group P (positive control), rats underwent gingivectomy and were given povidone iodine gel 10%.
- Group L, rats underwent gingivectomy and were given lemongrass periodontal dressing.
- Group M (positive control), rats underwent gingivectomy and were given a cellulose-based periodontal dressing with myrrh as active agent.

The animals were anesthetized with an intraperitoneal injection of ketamine and xyla mixture, then a gingivectomy with external bevel excision was made on anterior area of lower jaw in the labial surface of central incisive, exposing a rectangular area of 5 mm × 3 mm, using a sterile stainless steel surgical blade no 15 (Aesculap AG, Tuttlingen, Germany), and subsequently gingiva was separated by periosteal elevator. Each group received allocated regimen immediately after the wounding procedures by researcher's assistant. The animals were sacrificed with euthanasia and exsanguination, three rats for each stage of assessments, that is, at days 4, 7, and 14 after gingivectomy.

### Histological Assay

Tissue specimen were excised and immersed at days 4, 7, and 14 after gingivectomy in alcohol (70, 80, 90, 95, and 100%, respectively) and cleared three times in xylol, for 1 hour for each cycle. Paraffin was infiltrated to the specimen in three cycles, each cycle was performed for 1 hour. Specimens were embedded in liquid paraffin, blocked and sectioned with 5 μm using a microtome. These sections were then stained with hematoxylin and eosin (H&E) for fibroblast assessment, while Masson's trichrome staining was employed to reveal collagen deposition, then mounted with cover glass and glued with Entellan. The stained samples were viewed under electron microscope (Nikon Eclipse 80i) at ×40 magnification in five fields of view.

### Statistical Analysis

All data were subjected to statistical analysis using SPSS 20.0 (SPSS, IBM, New York, New York, United States). To test the normality of the data, Shapiro–Wilk test was employed, and all data were normally distributed. All values were represented as means ± standard deviation (SD) and were analyzed using one-way analysis of variance (ANOVA) followed by Tukey's post hoc for multiple comparisons to measure specific differences between pairs of means. A *p* -value of ≤0.05 was considered statistically significant.

## Results

All 30 rats survived the surgical procedures with no complications. Findings on each group were evaluated and histological differences were compared between control and experimental groups of section.

**Fig. 1:**
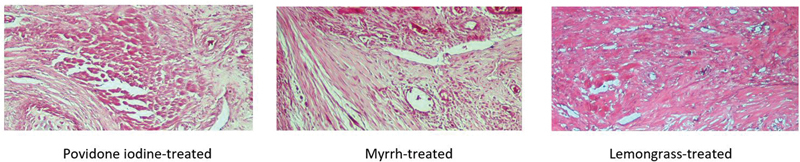
Histological assessment of fibroblast density among groups at day 4 (hematoxylin and eosin [H&E], ×40). Fibroblast activity was better expressed in lemongrass group than in myrrh and povidone iodine group ( *p* < 0.05).

The growth of fibroblasts and collagen deposition were calculated in days 4, 7, and 14 after gingivectomy. On day 4 following gingivectomy, the comparison of the histological findings demonstrated that in the group L, fibroblast activity was better expressed than in groups M and P ( *p* ≤ 0.05; Table 1 ). Tukey's post hoc analysis revealed that there were significant differences in fibroblast between groups L and P, and between groups L and M with no significant difference between groups P and M ( Table 2 ). Histological assessment showed fibroblast density among groups at day 4 ( Fig. 1 ).

**Table 1:** Fibroblast proliferation on day 4

Group	Number of fibroblast		*p* -Value
	Mean	SD (±)	
P	72.4	5.6	
L	105.8	17.8	0.038 a
M	72.5	14.5	

Abbreviations: L, lemongrass; M, myrrh; P, povidone; SD, standard deviation.

a Statistically significant, analysis of variance.

**Table 2:** Tukey's post hoc analysis on fibroblast on day 4

Group	Group	Mean difference	SE	*p* -Value
P	L	33.40000	11.16980	0.05 a
L	M	33.3333	11.16980	0.05 a
M	P	0.06667	11.16980	1.000

Abbreviations: L, lemongrass; M, myrrh; P, povidone; SE, standard error.

a Statistically significant.

**Fig. 2:**
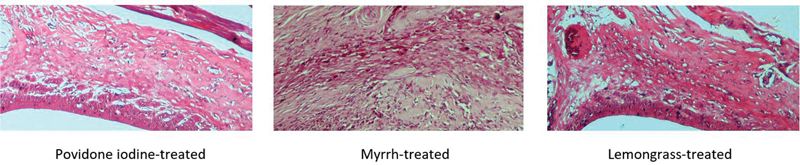
Histological assessment of fibroblast density among groups at day 7 (hematoxylin and eosin [H&E], ×40). Fibroblast in lemongrass group was greater compared to myrrh and povidone iodine group ( *p* < 0.05).

On day 7 following gingivectomy, group L has the most fibroblast proliferation compared with groups P and M ( *p* < 0.00). The number of fibroblasts in group P was fewer, whereas in group M was moderate and group L had the greatest ( Table 3 ). The Tukey post hoc showed that fibroblast in group L was greater compared with groups P and to M ( Table 4 ). Fig. 2 showed histological assessment of fibroblast density among groups at day 7.

**Table 3:** Fibroblast proliferation on day 7

Group	Number of fibroblasts		*p* -Value
	Mean	SD (±)	
P	51.6	26.9	
L	281.5	11.9	0.000 a
M	116.6	22.7	

Abbreviations: L, lemongrass; M, myrrh; P, povidone; SD, standard deviation.

a Statistically significant, analysis of variance.

**Table 4:** Tukey's post hoc analysis on fibroblast on day 7

Group	Group	Mean difference	SE	*p* -Value
P	L	229.9	17.55052	0.000 a
L	M	164.9	17.55052	0.000 a
M	P	65.0	17.55052	0.023 a

Abbreviations: L, lemongrass; M, myrrh; P, povidone; SE, standard error.

a Statistically significant.

**Fig. 3:**
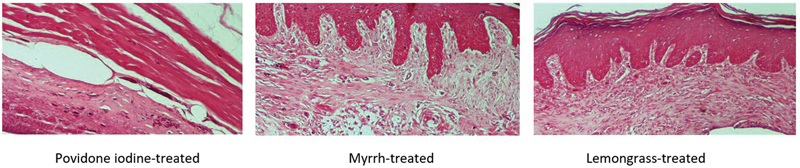
Histological assessment of fibroblast density among groups at day 14 (hematoxylin and eosin [H&E], ×40). It was shown that epithelium in povidone iodine group had started to form, while in myrrh and lemongrass group, the epithelium was better developed, with a greater number of layers, and even slight keratinization.

**Fig. 4:**
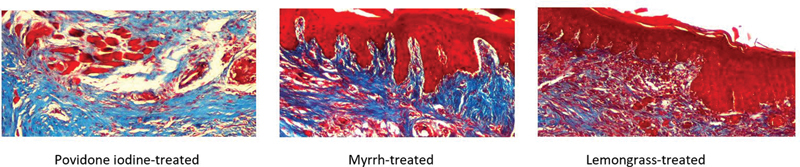
Histological assessment of collagen deposition among groups at day 14 (Masson's trichrome, ×40). Increased formations of new collagen was observed in all groups although the differences between groups were not statistically significant ( *p* = 0.868).

On day 14 following gingivectomy, the comparison of the histological findings demonstrated that epithelium in group P had started to form, while in groups L and M, the epithelium was better developed with a greater number of layers and even slight keratinization ( Fig. 3 ). Fibroblast number in group P became the highest ( Table 5 ), the Tukey post hoc was then employed to find the comparison between pair groups, and it was shown that no significant differences between groups L and M ( Table 6 ). Means of fibroblasts among groups on days 4, 7, and 14 were significantly different ( Table 7 ). Increased formations of new collagen were observed in all groups, although the differences between groups were not statistically significant ( *p* = 0.868; Table 8 ). Fig. 4 displayed histological assessment of collagen deposition among groups at day 14.

**Table 5:** Fibroblasts proliferation on day 14

Group	Number of fibroblast		*p* -Value
	Mean	SD (±)	
P	187.5	11.5	
L	62.6	27.7	0.001 a
M	102.2	14.2	

Abbreviations: L, lemongrass; M, myrrh; P, povidone; SD, standard deviation.

a Statistically significant, analysis of variance.

**Table 6:** Tukey's post hoc analysis on fibroblast on day 14

Group	Group	Mean difference	SE	*p* -Value
P	L	124.9	15.65016	0.001 a
L	M	39.6	15.65016	0.098
M	P	85.3	15.65016	0.004 a

Abbreviations: L, lemongrass; M, myrrh; P, povidone; SE, standard error.

a Statistically significant.

**Table 7:** Mean of fibroblast among groups on days 4, 7, and 14

	P	L	M	*p* -Value
Day 4	72.5	105.8	72.5	0.038 a
Day 7	51.6	281.5	116.6	0.000 a
Day 14	187.5	62.6	102.2	0.001 a

Abbreviations: L, lemongrass; M, myrrh; P, povidone.

a Statistically significant, analysis of variance.

**Table 8:** Collagen deposition on day 14

Group	Number of collagen		*p* -Value
	Mean	SD (±)	
P	58,741.3	36,098.3	
L	71,845.3	19,414.1	0.868
M	68,916.7	35,370.0	

Abbreviations: L, lemongrass; M, myrrh; P, povidone; SD, standard deviation.

Note: Analysis of variance, not significant.

## Discussion

The present study was conducted to evaluate the effect of lemongrass wound dressing on the healing process after gingivectomy in rats at histological level. In wound healing, good tissue growth was defined as a tissue rich in fibroblasts and dense in newly synthesized collagen determined by Masson's trichrome. 26

To the best of our knowledge, no studies have been performed to evaluate the effect of lemongrass extract incorporated in wound/periodontal dressings. The findings of this present study revealed that on days 4 and 7, the rate of fibroblast proliferation of the wound was significantly superior following the application of lemongrass periodontal dressing compared with other groups. Fibroblasts are the main source of extracellular matrix protein, mostly collagen and fibronectin, that constitutes the newly formed granulation tissue, thereby providing structural integrity to the wound. 27 In wound healing, an inflammatory phase initiates the process, where platelets, neutrophils, macrophages, and lymphocytes migrate to a wound. Subsequently, the proliferative phase shows an increase in fibroblasts and macrophages with a decrease in the acute-phase reactants. The final phase is a remodeling phase where fibroblasts assist in the reconstruction of the extracellular matrix and deposit collagen. It can be observed that epithelial cells start to migrate to the borders of the lesion, and to form the new epithelium, while fibroblasts continue to proliferate during the first few days postsurgery. 28 29 Therefore, fibroblasts play a significant role in the healing of tissue trauma, including surgical wounds, as well as in epithelization and collagen synthesis. 30 31 Our findings showed that fibroblast activity was greater in the lemongrass group, suggesting that lemongrass pack has a positive effect on wound healing. Furthermore, in lemongrass and cellulose-based pack groups, epithelium was better developed, indicating that the proliferation phase characterized by epithelial proliferation had occurred. This result is in agreement with a study conducted by Primasari and Sinulingga which observed an increase in the thickness of the oral mucosal epithelium after administration of lemongrass extract in buccal part of rats following incisions. 32

We used povidone iodine gel and a cellulose-based dressing containing myrrh as control, since povidone iodine is considered to have the broadest spectrum of antimicrobial action and rapid antiviral and antifungal activity. The mechanism of action of povidone iodine is attributable by the active moiety (non-Polyvinylpyrrolidone [PVP]-bound “free” iodine) being released into solution from the PVP-I complex, penetrating the cell wall, and inactivating cells by forming complexes with amino acids and unsaturated fatty acids, resulting in impaired protein synthesis, and alteration of cell membranes. 33 While myrrh (a mixture of volatile oil, gum, and resin) is a popular herbal compound that has been commonly used to treat a variety of inflammatory conditions for centuries, it has been shown to be biocompatible with oral tissues and promotes healing by promoting earlier remodeling. 34 The healing properties of myrrh may be explained by its induction of maturation and activation of leucocytes. 35

Periodontal dressing is placed to protect oral wound against mechanical injuries during mastication and against bacterial invasion into the tissue, with the aim to decrease pain and facilitate healing, without any adverse effects such as increase formation of bacterial plaque, bacterial resistance, cytotoxicity, delayed wound healing, or disturbed occlusion. Furthermore, the ease of use is also one consideration of choice. Cellulose-based periodontal pack is soft and unlike conventional periodontal dressings, it remains elastic at all times, it needs no mixing in preparation and slowly gets dissolved over a period of 2 to 3 days, hence no removal is needed. It is suggested that incorporating lemongrass extract in a cellulose-based matrix, may enhance the biological properties of this resorbable pack.

Several studies had evaluated the effect of herbal active compounds incorporated in wound dressing. Faiga et al studied the effect of a mixture of *Curcuma longa* rhizome extract and zinc oxide–eugenol (ZOE) dressing on angiogenesis, compared with ZOE dressing alone. The addition of *C. longa* rhizome extract showed added value in the wound healing process in term of the number of neovascular. 36 Nevertheless, the wound sites examined were in the dorsal part of rats which had different environment and circumstances from oral sites. In the present study, wounds were created in the gingival tissue surrounding by oral environment. Unlike skin surface, wound healing in the oral cavity occurs in the presence of many challenges, including a high bacterial and viral load and cannot be sterilized from bacterial plaque formation. Therefore, wound surface must be protected from the external environment or infection after oral surgery since infection keeps a wound in an inflammatory state and may lead to a delayed healing.

Within the wound healing process, the inflammatory phase involves homeostasis and inflammation that start at the moment of injury and continue for up to 4 to 6 days. The proliferation phase involves epithelialization, angiogenesis, granulation tissue formation, and collagen deposition, and takes place from days 4 to 14 after injury. Epithelial-cell migration starts after 24 hours.

We had also observed a beneficial effects of *Moringa oleifera* Lam leaves extract incorporated in a cellulose-based wound dressing on wound healing in palatal rats, but the applications of the dressing were more frequent (once a day in 14 days) which clinicians or patients were not in favor of that. 37 In the present study, the application of lemongrass periodontal pack was only once immediately after wounding and no repetition was done which is easier and favorable, and we found that one time application is able to accelerate wound healing following rat's gingivectomy.

Lemongrass has already known for its beneficial effects in oral health. As a folk medicine, based on empirical studies, it is commonly used as remedies for gum swelling, periodontitis (inflammation of tooth supporting tissue), and tooth ache by removing bacteria from the oral cavity. 38 It is interesting to note that *in vitro* study showed that lemongrass indeed possess antimicrobial activity against several oral pathogens. Rêgo et al investigated that essential oil of lemongrass was effective in controlling bacterial growth in biofilms of *Streptococcus mutans* , the main causative agent of tooth decay. 39 Furthermore, it was shown that lemongrass essential oil has remarkable antimicrobial and antibiofilm activities against the dental plaque bacteria. 40 The oral cavity harbors over 700 different species of bacteria and is described as one of the most intricate ecosystem. Though the majority of them are considered as commensally, some of them are responsible for oral infections ranging from tooth decay to periodontal diseases and gingiva related infections. 41 That is why wound healing located in oral sites will always have sustainable challenge from bacteria residing in this environment. In another study, lemongrass essential oil was able to inhibit the growth of putative periodontal pathogens, especially *Aggregatibacter actinomycetemcomitans* , *Actinomyces naeslundii* , and *Porphyromonas gingivalis* , as well as the tetracycline-resistant strains. The results showed the superior properties of the essential oil and suggested the use of the oil with other antibiotics against resistant bacteria. 42 43 44 The mechanism of action suggested is that lemongrass oil and its components induced intracellular leakage, as well as ultrastructural changes in bacterial cells, as it was shown by electron micrographs that a hole occurring on the surface of bacterial cell treated with citral, a main component of lemongrass, at a concentration of 4 μL/mL. 45

Another beneficial effect of lemongrass essential oil is its anti-inflammatory, as well as antifungal, properties. The exact mechanism of the anti-inflammatory effect of lemongrass is unclear. However, it has been suggested that several molecules contribute to the partial inhibition of the release of inflammation mediator substances. 46 Citral, geranial, neral, and carvone, the main components of lemongrass, have been shown to inhibit the production of interleukin (IL)-1b, IL-6, and tumor necrosis factor (TNF)-α. 47 48 Moreover, lemongrass was shown to have antioxidant activity and is able to scavenge free-radical molecules. 49 In a study evaluating lemongrass aqueous extract, pholyphenolic compounds found in lemongrass, that is, rutin, isoquercetin, catechin, and quercetin were able to reduce the atherogenic index and enhances the serum antioxidant capacity in rats. 50 Wound healing implicates inflammation in the phases. Inflammation is a double-edged sword, having both favorable and unfavorable consequences and during inflammation phase, leucocytes produces oxygen radicals as oxygen-dependent mechanisms. These free radicals are excreted as a weapon to kill bacteria but also may have detrimental effect to the body cells. An excess of free radicals and/or a depletion of antioxidants may result in oxidative stress leading to cellular dysfunction and sometimes cells' death. In this circumstance, antioxidants help to protect cells from damage which is caused by free radicals produced during inflammation. 51

## Conclusion

In conclusions, within the limitations of the present study, the following inferences can be drawn: (1) lemongrass extract incorporated in resorbable periodontal dressing was able to stimulate fibroblast and collagen deposition during the initial phase of wound healing after gingivectomy in rats, and (2) one-time application of lemongrass pack immediately after gingivectomy was sufficient to protect wound site and accelerate healing. However, further studies are required to clarify the optimal concentration and physical stability before its clinical application.
